# “How Do We Start? And How Will They React?” Disclosing to Young People with Perinatally Acquired HIV in Uganda

**DOI:** 10.3389/fpubh.2017.00343

**Published:** 2017-12-13

**Authors:** Stella Namukwaya, Sara Paparini, Janet Seeley, Sarah Bernays

**Affiliations:** ^1^Medical Research Council (MRC), Uganda Virus Research Institute, Entebbe, Uganda; ^2^Anthropology and Sociology of Development, Graduate Institute of International and Development Studies, Geneva, Switzerland; ^3^Global Health and Development, London School of Hygiene & Tropical Medicine, London, United Kingdom; ^4^Sydney School of Public Health, Sydney Medical School, University of Sydney, Sydney, NSW, Australia

**Keywords:** children and adolescents, young people, disclosure, caregivers, HIV

## Abstract

Despite great advances in pediatric HIV care, rates and the extent of full disclosure of HIV status to infected children remain low especially in resource-constrained setting. The World Health Organisation recommends that, by the age of 10–12 years old, children should be made fully aware of their HIV-positive status. However, this awareness is often delayed until much later in their adolescence. Few studies have been conducted to investigate what influences caregivers’ decision-making process in this regard in low-income settings. In this article, we present an analysis of care dyads of caregivers and HIV-positive young people in Kampala, Uganda, as part of the findings of a longitudinal qualitative study about young people’s adherence to antiretroviral therapy embedded in an international clinical trial (BREATHER). Repeat in-depth interviews were conducted with 26 young people living with HIV throughout the course of the trial, and once-off interviews with 16 of their caregivers were also carried out toward the end of the trial. In this article, we examine why and how caregivers decide to disclose a young person’s HIV status to them and explore their feelings and dilemmas toward disclosure, as well as how young people reacted and the influence it had on their relationships with and attitudes toward their caregivers. Caregivers feared the consequences of disclosing the young person’s positive status to them and disclosure commonly occurred hurriedly in response to a crisis, rather than as part of an anticipated and planned process. A key impediment to disclosure was that caregivers feared that disclosing would damage their relationships with the young people and commonly used this as a reason to continue to postpone disclosure. However, young people did not report prolonged feelings of blame or anger toward their caregivers about their own infection, but they did express frustration at the delay and obfuscation surrounding the disclosure process. Our findings can inform the ways in which mainstream HIV services support caregivers through the disclosure process. This includes providing positive encouragement to disclose fully and to be more confident in initiating and sustaining the timely process of disclosure.

## Introduction

More than two million young people below the age of 15 years are living with HIV globally with the vast majority in sub-Saharan Africa (1). Over the years, there have been remarkable improvements in providing access to pediatric HIV treatment with antiretroviral therapy (ART), which has significantly reduced mortality rates (2) and enabled perinatally HIV-infected young people to live through adolescence and into young adulthood (3). Despite great advances in pediatric HIV care, however, rates and the extent of full disclosure of HIV status to HIV-positive young people remain low especially in resource-constrained settings (4).

Young people with perinatally acquired HIV often start ART in early childhood before knowing why they are taking it (5). The need to start treatment in childhood is acute, and at that time it may be accepted that the child will be told their HIV status and the reasons for their ART when they are older. The most recent global guidelines from the World Health Organisation (WHO) (6) on the disclosure to young people living with HIV recommend that once children are of school-going age, and certainly by the age of 10–12 years old, they should be fully made aware of their condition and its consequences for them. However, this awareness is often delayed until much later in their adolescence (7–9).

In Uganda, it is estimated that of the 1.2 million people living with HIV, 13% are young people under 15 years (10). There are over 600,000 people in Uganda with access to ART and about 43,000 of these are young people below the age of 15 years (11). However, a study on disclosure of HIV status to young people between 5 and 17 years in Southwestern Uganda showed that only 31% of the young people had been informed that they had HIV (12). Sociocultural norms are likely to influence disclosure conversations. For example, in Uganda discussing the routes of HIV transmission with young people as it relates to sexuality and the sexual behavior of their parents is problematic (13, 14). Despite this, few studies have been conducted to investigate what influences caregivers’ decision-making process in this regard in low-income settings.

In this article, through an analysis of care dyads of caregivers and HIV-positive young people, we examine why and how caregivers’ in Kampala, Uganda, decide to disclose a young person’s HIV status to them, as well as exploring their feelings and dilemmas toward disclosure.

### The Importance and Challenges of Disclosure: Current Views from the Field

Young people who have been appropriately informed of their illness early, exhibit better coping skills and fewer psychosocial problems (15). Being aware of their own HIV status as they transition into adolescence is crucial for young people living with HIV to assume some responsibility for managing their own treatment (16). Most available evidence shows an association between disclosure and improved adherence to treatments (4, 17) although a recent qualitative synthesis suggests that the relationship between disclosure and the development of positive adherence habits is mixed (18). Beyond adherence, pediatric HIV disclosure is also positively associated with safer sex behaviors in adolescents, enabling them to actively participate in making decisions about their health and sexuality (19, 20).

The HIV disclosure literature conceptualize disclosure as a process and recommend that the disclosure responsibility lies with the caregivers/guardians of the child and that it should be tailored to a child’s cognitive development (21). These notions are reflected in the most recent WHO (22) and also the national guidelines from the Ministry of Health in Uganda (23). However, disclosure of perinatally acquired HIV is of course a complex and challenging process for caregivers and health-care workers. Indeed, research in African settings, including Uganda, has demonstrated that caregivers are reluctant to disclose and this is manifested in disclosure being postponed, treated as a one-off event and, when done, being partial and incomplete (8, 24, 25). Partial disclosure is the term used to describe situations in which young people are given some but not all the information about their illness or condition. They may be made aware of the fact that they have a health condition which requires them to take lifelong medication without being told that their “condition” is HIV (26).

Reasons for caregivers’ non-disclosure of young people’s HIV status cited in the literature include the pernicious stigma associated with HIV, which leads to caregivers being afraid that, once informed, young people may disclose their individual HIV status to others, placing them at risk of being discriminated against, for example, in schools (24). Caregivers’ postponement of disclosure may also stem from their worries about their child’s cognitive abilities and emotional readiness to receive the news of their (young people’s) own HIV-positive status (27). Fears that young people would be emotionally affected, cry, be sad, and give up on life following disclosure have all been identified as barriers to disclosure (28, 29). A study among Ugandan caregivers has shown that not disclosing to young people is seen as a form of protection from anticipated stress for the young people (9).

Caregivers may also have doubts as to the right timing for disclosure and about how much information about HIV is meant to be shared with young people (30). Indeed, several studies have reported on caregivers’ perceived lack of skills on how to disclose HIV infection to children (30, 31), for example, not knowing how to talk to the young people about HIV or how to explain mother to child transmission, which may lead them to engage in partial disclosure (32).

Furthermore, the inherited nature of the illness with perinatal acquisition means that there are direct risks for the caregivers and related household too once a young person’s status becomes known (24). Thus, as Muparamoto and Chiweshe (33) have shown, caregivers’ decision to disclose is affected by complex expectations in which they attempt to control the “strategic event” of disclosure to minimize the potential damage to their own identity and that of their child’s. However, another recent study in Uganda with young people aged 13–17 years has shown that they may exhibit considerable resilience in response to HIV disclosure (34), so there may be a disconnect between caregivers fears and young people’s response.

When disclosure does happen, there is significant evidence to suggest that caregivers decide to disclose on the instruction from health-care workers in a bid to support young people’s adherence to HIV medication, while unintentional or forced disclosure has also been shown to be common (9, 35). Caregivers may also disclose as a result of young people’s persistent questioning about why they are taking medication, if they will ever stop and when they will get better (8).

Current global and local literature thus suggests that, despite policy guidance, caregivers hesitate to disclose HIV diagnosis to the young people. With this article, we aim to compare available literature and our own qualitative study findings from Uganda, to contribute to a better understanding of local dynamics pertaining to the Ugandan context and to illuminate caregivers *as well as* young people’s perspective on disclosure. We consider how these experiences impact on the disclosure process, how disclosure is received by young people themselves, and what can be done to improve support to caregivers to be more confident in initiating the timely process of disclosure. This will be important in the development of interventions to support caregivers and HIV-positive young people through a process of fuller and ongoing disclosure.

## Materials and Methods

### Study Setting and Population

This was a longitudinal qualitative study, involving young people perinatally infected with HIV and their caregivers, that was conducted between 2011 and 2016 at the Pediatric clinic of the Joint Clinical Research Centre (JCRC) in Kampala, Uganda. JCRC provides comprehensive HIV/AIDS care and management to about 2,000 HIV-infected young people and over 150,000 adults. It was the first HIV/AIDS treatment center in Uganda to provide ART and is currently the country’s only reference center for third-line therapy. The study was embedded within a clinical trial (36) (BREATHER) which was testing the efficacy of a treatment interruption intervention, Short Cycle Therapy on efavirenz-based regimens (5 days on, 2 days off treatment) for young people (8–24 years) living with HIV (37).

Twenty-six young people were recruited purposively to participate in repeat in-depth interviews, audio diaries, and focus group discussions. The topics covered in this article were not discussed in the focus group discussions or the audio diaries and so only the data from the in-depth interviews will be presented here. Purposive sampling was carried out to increase the likelihood of capturing various experiences. Young people were eligible to participate in the study if they were aged 10–24 years and had full knowledge of their HIV status for at least 6 months before being enrolled in the trial. The assumption is that 6 months after disclosure, through continued counseling and support, young people would have been more likely to have understood many of the implications of their HIV-positive status. A minimum age of 10 years was selected to ensure that participants had the cognitive abilities to meet the broader aim of the study. Young people who met the criteria were approached for study participation together with their caregivers (for those below 18) within the waiting area of the clinic during their scheduled clinic visit. They were then taken to a private room in the clinic where they were given a detailed explanation of the study after which they gave written informed consent for participation. For those below the age of 18 years, assent was obtained after their caregivers had provided written informed consent for them to participate in the study.

We understood consent to be a process which ran from initial recruitment through to dissemination, in which both caregivers and young people were involved. Study participants were given the opportunity to speak to a counselor, based in the clinic, at any time that they needed. The study provided additional funds to cover the counselor’s time, so that this resource would always be available throughout the study. This was in line with best research practice in this context, offering an integrated and sustainable mechanism for support. All young people were offered the opportunity to speak to a counselor in case they needed to after they were interviewed, but none chose to do so. Also, each time a participant visited the clinic to engage in the qualitative study their transport costs were refunded, again in line with best local research practice.

Toward the end of the trial, all the 26 participants were asked to nominate a significant other, who was someone acting in a primary caring role for them, and that they would be comfortable for us to invite to also participate in the study. To be included in the study, the caregiver needed to be able to give all required information especially with regard to the child’s illness trajectory. To manage the volume of data, we had set a predetermined sample size of 16 caregivers. Once a young person had given their specific verbal consent, 16 caregivers were purposively selected and approached through their registered phone contacts at the clinic and invited to participate in an individual in-depth interview, and there were no refusals. The purposive sampling criteria ensured maximum variation within our sample for factors such as gender, socioeconomic background, and relationship to the child among others. A detailed explanation of the study including purpose, procedure, rights of volunteering participants, and assurance of confidentiality and anonymity were given to the caregivers. They provided informed consent.

The rationale for including the caregivers in the study was the recognition that they play a significant role in young people’s lives and influence their understanding and experience of living with HIV. We wanted to understand how caring for a young person living with HIV was understood and perceived, as well as learn more about whether and how the caregivers’ perceptions of HIV might shape their caring and relationship with the young person.

We present the findings from these care dyads, drawing on the 16 caregiver interviews and the in-depth interview data from the related young people. We deliberately do not present matched dyad data so that the caregivers and young people are not able to identify each other from the information we present here.

### Data Collection and Analysis

Audio recorded, in-depth semi-structured interviews were conducted using a topic guide, which was tailored to the circumstances of each individual. The topic guide covered the following key areas of investigation: managing children’s adherence; understandings of care; relationships within the household; and disclosing HIV status to young people. The guide was adjusted according to the circumstances that the caregiver disclosed, for example, whether they told us that they were themselves HIV positive. Interviews were conducted in English or the local language (Luganda) according to the participant’s preferred language of choice to ensure confidence in their responses. Each interview session lasted between 45 and 60 min. Stella Namukwaya, a social scientist with extensive training and years of experience in qualitative research, conducted the interviews.

Recorded interviews were transcribed verbatim and translated where necessary by the first author. Discussions were held with other members of the research team (coauthors on this article) after each of the interviews to identify the emerging themes and to refine the interview guide to ensure issues arising were exhaustively explored.

Thematic analysis was carried out by all the four members of the research team (Stella Namukwaya, Sara Paparini, Janet Seeley, and Sarah Bernays). Transcripts were read and re-read to identify emerging patterns with specific focus on disclosure of HIV to young people. Themes were developed from participant’s responses and categorized as shown in Table 1. Content theme analysis was done to ensure that all relevant information was grouped and coded appropriately. Inter-rater reliability was very high (more than 80%) and the few discrepancies that arose were discussed and reconciled during regular weekly team meetings. Pseudonyms are used in this article to protect confidentiality.

**Table 1:** Themes and subthemes.

Theme	Subtheme
Reasons for caregivers’ reluctance to disclose	Fear of mentioning HIV
	Fear of psychological damage to child
	Fear of damaging child–caregiver relations
	Concerns about stigma
	Concerns about Discretion, HIV Stigma, and Discrimination
	Not knowing how to talk about HIV

Factors that motivated disclosure	Adherence crisis/importance of drugs
	Young people’s curiosity

Young people’s reactions	Temporary feelings of shock. Frustration about delays in being disclosed to and partial nature of disclosure

### Ethical Clearance

The study received institutional and national ethical approvals from the Uganda National Council for Science and Technology, National Drug Authority and the Joint Clinical Research Centre Institutional Review Board.

## Results

### Participant Characteristics

A total of 16 caregivers took part in this study and of those interviewed, the majority were women (13 out of 16). Four of them were biological parents while the remaining were other relatives such as aunts, an uncle, stepmothers, and grandparents. Most caregivers were reportedly HIV negative. Of the 26 young people in our study, only 3 had primary male caregivers who brought them to the clinic and whom the young people nominated to be selected to participate in the study. All three were included in this study. This reflects common gender pattern of caregivers’ accompanying young people to this clinic. Discussion about the caregiver’s status only occurred when initiated by the caregiver themselves. We did not ask them directly about their HIV-positive status, however, all the caregivers talked about their status during the course of the interviews. Most caregivers were making a living on irregular, small scale business initiatives such as hairdressing and selling in markets. The majority had attained primary education while the rest completed secondary or vocational education.

All of the 26 young people who participated in the qualitative study had acquired HIV vertically and many had lost one or both parents. There were 14 girls and 12 boys in the study, between the ages of 10 and 24 years, and most of them were attending or had attained secondary school education. Further details of the sample can be found in our other publications from this study (7, 36, 38).

Although in the relevant guidelines responsibility to disclose HIV diagnosis is understood to lie with caregivers, we found that caregivers were very reluctant to do so. In this article, we first look at the reasons, which prompted caregivers to disclose and their concerns in doing so. We then present findings from the young people’s interviews to explore whether the concerns of the caregivers are borne out in young people’s narratives around finding out about their own HIV status.

### Reasons for Caregivers’ Reluctance to Disclose

Caregivers faced various difficulties in initiating disclosure conversations and talking to the young people about HIV and, where relevant, AIDS-related illness, and employed strategies to postpone disclosure. In the interviews, they provided many, often interlinked explanations for their decisions to delay conversations.

#### Fear of Mentioning HIV

Breaking news about HIV was something caregivers wished to avoid as long as possible. They did this by fabricating alternative explanations as to why their children were taking daily drugs. Almost any condition was considered preferable to HIV, so care-givers commonly told the young people that they were taking medicines for kidney disease, malaria or tuberculosis, for example. They did not necessarily presume that this was a strategy that would work indefinitely, but caregivers reported cycling through a range of alternative explanations:

In the beginning, she didn’t know what she was suffering from, they (father) first told her that she had kidney disease, then later on that it was malaria (Kitty’s stepmother).

I would tell her that she had TB and even when we both started taking ART I told her that they were drugs for TB (Beth’s mother).

#### Fear of Emotional and Psychological Damage to the Child

When asked why they were avoiding disclosure, caregivers explained their reluctance was based on their anxiety about the unknown potential psychological outcomes of disclosure for children and young people. Caregivers’ reported being worried that disclosure might result in young people withdrawing from active social and educational engagements and interactions. They feared it would deprive them of their happiness and of the opportunity to live what the caregivers considered to constitute “a normal life.” As Tessa’s caregiver explained:

I was so scared of how she would react. I thought that she might have regrets, feel sad, start to isolate herself from people and feel like she has a problem (Tessa’s aunt).

Among our sample, the fear of the repercussions from disclosure was expressed more strongly by women than by men. Two of the male caregivers reported that even though they were concerned about the child’s emotional well-being they were more confident that the young people would not be grossly affected and would instead build resilience and understanding once their illness had been disclosed to them. However, male caregivers disclosed to the young people even later than the women. Men reported that they wanted to do this once the young people had reached the age of 13 years, and justified this by saying they were waiting for the young people to “mature” because then they would cope better with the information and not be damaged by it. However, with only three male participants in our study, any gender-related significance of this finding should not be overinterpreted.

I told (disclosed) Leah when she was 13 and I knew that it would be easy because in the end she would have understood (Leah’s father).

Concealment and delay strategies were additionally entwined with a desire to protect the young people from worrying about whether their caregivers or siblings were also HIV positive, and under any kind of threat:

We refused to tell them because we didn’t want to scare them because they would think that we also have HIV (Amy’s caregiver).

#### Fear of Damaging Child–Parent Relationships

For biological caregivers, disclosure carried with it more directly personal risks too. They were also concerned that young people would ask question as to how they had become HIV positive, which would lead to uncomfortable conversations about transmission, the parents’ own experience of becoming HIV positive and questions of loss with regard to HIV-related deaths in the family. Mothers, in particular, were worried about being seen as “the source of infection” and blamed by the child. They were worried that disclosing would damage their relationship with their child, with young people being angry with them for “burdening” them with the virus.

For all caregivers, there was an anxiety that the young people had been failed by their parents by not being able to adequately protect them from acquiring HIV. In reality, as the caregivers were looking after young people aged 10–24 years old, there had been significant changes in provisions to prevent mother to child transmission since most of these young people had acquired HIV at birth, with many of them not able to benefit from current preventive treatments and practices.

Nearly all of the caregivers spoke about the risks that disclosure could potentially result in conflict and also put a strain on family relationships. This highlights the concern over the wave of revelations that disclosure to their child might precipitate.

I was worried and didn’t know how to tell her (Child) that I got HIV from her father, because that was what killed him (Jules’s mother).

#### Concerns about Discretion, HIV Stigma, and Discrimination

For both biological and non-biological caregivers, their desire to protect the young people from the potential for HIV stigma and discrimination further hindered disclosure. Caregivers did not trust that, once told, their child would be able to keep their own status a secret. They rationalized that by concealing this information from the young people and thereby limiting the numbers of people who got to know about young people’s HIV status, they were protecting them from negative outcomes like being a source of gossip and being rejected by friends:

The disadvantage is that if you tell them, they will also tell someone else so people might start to gossip about the child … many people don’t know that he is sick (Gucci’s aunt).

This concern persisted despite the low levels of direct discrimination that the caregivers had themselves experienced or witnessed. Only one of the caregivers reported being exposed to HIV-related discrimination. But they were all aware of stories about how people living with HIV had been subjected to discrimination in the past and presumed that this prevailing attitude continued and could affect the young people.

The caregivers considered that adults were far better placed to deal with any negative fallout from their status becoming known. By contrast, they perceived that children were not competent to manage their own secrets. They stressed that stigma was a serious risk and felt that, if young people had to be subjected to it, the consequences would be too heavy for them to bear. Hence, even though they would agree that there might be benefits from disclosing one’s HIV status, such as support from peers, they were quick to emphasize that these only applied to adults living with HIV.

Delaying disclosure until young people were considered competent in managing information formed an important part of a more general strategy of maintaining silence about HIV. In most cases, caregivers had kept the HIV status of the child from other household members, and biological parents had often kept their own HIV status a secret from some or all of the rest of the household, too. So not talking to young people about HIV served also as an indirect way for caregivers to control information about themselves and their household.

#### Not Knowing How to Talk about HIV (Inadequate Language and Understanding)

Caregivers also described feeling woefully underprepared to initiate and address disclosure conversations with the young people. They lacked confidence in how they should do it. They described not being certain about their biomedical knowledge of HIV and felt what they did know was insufficient to facilitate the disclosure process:

We used to think about it (disclosure) a lot and every time we tried to we would just postpone it (Max’s Aunt).

Although common overall, this was particularly an issue for non-biological caregivers who may have had little knowledge of HIV compared with biological caregivers that had been living with HIV themselves or those who had close experience of their partners being affected. We found that biological caregivers who were themselves living with HIV had usually been able to disclose at home, while many of the non-biological caregivers had required the presence of counselors or other health-care staff because they did not know what to say.

This lack of confidence appeared to be even more problematic once the caregiver needed to move beyond naming the child’s condition. They described that their lack of knowledge and skills to manage conversations about HIV treatment, transmission, and prevention severely inhibited their capacity to engage in further discussions. An example many caregivers gave was not knowing how to explain changes in prevention of mother to child transmission (PMTCT) to young people. As reported in the interviews, caregivers’ explanations of transmission were commonly inaccurate and invariably brief, as exemplified by Lisa’s Aunt:

I explained to her that maybe it (HIV infection) was because of the c–section so that is how she got infected (Lisa’s Aunt).

Some caregivers also expressed concern about whether their child would be able to have sexual relationships and have children in the future. Although this was not an issue around the time of disclosure, but came up much later in adolescence, such concern also highlighted in caregivers’ interview accounts their limited understanding of PMTCT and HIV in general. It also fueled their concerns about their capacity to respond adequately to any of the young people’s questions upon being disclosed to and their anxiety about the psychosocial impact of being told.

Some non-biological caregivers also lacked biographical knowledge pertaining to the child’s acquisition of HIV as they had taken on caring responsibilities after the death of the child’s parent. So, as they might not have been explicitly told how the child had acquired HIV this ambiguity added an additional layer of complexity. But it was also used to justify the partial approach to disclosure which deliberately avoided moving beyond naming their condition.

### Factors That Motivated Disclosure

When caregivers did disclose, it very often tended to be because circumstances necessitated it. The decision was also never taken or planned for by the caregiver alone, but in response to the pressure of events. Thus, it was not always a decision based on what might be considered “age-appropriate” timing or the circumstances of the child.

#### Adherence Crisis and the Importance of Drugs

Supporting the child’s adherence to their treatment was the priority for many caregivers and a child struggling with their adherence was the most common circumstance in which a caregiver disclosed to their child their HIV status. This was because health-care workers frequently stressed to the caregivers that adherence would only improve if the child was made aware of their condition and thus more likely to understand how important the drugs were for their survival and well-being. Some explained that they would have had no intention of disclosing to young people until they were much older if they had been taking their medication well: “*If he had been adhering to his drugs, I wouldn’t have been bothered with that*” (Finn’s caregiver).

Of note, this approach highlights that many caregivers did not consider that disclosure was necessary beyond adherence and that children had an “independent” right to know their HIV status.

Focusing the disclosure conversation on the importance of drugs, however, was not only done because adherence was the key reason for disclosure in the first place. It was seen as a way for caregivers to comfort and give hope to the young people while revealing their status, to assure them that amidst their life-threatening condition there is a known solution: taking the drugs. It appeared to give caregivers, and by extension young people, some control over what might happen in the future:

I comfort her and tell her that there are people on drugs who are now twenty years old or even thirty who are able to study and complete University (Leah’s dad).

#### Young People’s Curiosity

In some cases, disclosure was triggered by young people’s persistent questions to their caregivers about why they were taking medicines every day, when or whether they would be able to stop taking them and whether they would ever be cured of whatever illness they had. Caregivers reported that as young people grew older (from the age of 10 years onward) they became increasingly dissatisfied by the explanations that they had been given. In some cases, for example, when they were the only person in the household that they knew to be taking treatment, children had to ask questions for a considerable time to push to eventually be given the answer.

In the beginning, she didn’t know what she was suffering from […] she started asking questions as she grew older because she was wondering why she was not getting better so they told her (Kitty’s stepmother).

She kept asking me why she was taking drugs when other children in the home were not (Tessa’s Aunt).

In households where pill taking was not such a guarded and secret activity, disclosure was also a response to questions from other household members. Household members, especially the younger ones, became curious about why other young people were taking drugs every day unlike them or others in the home. Daisy’s uncle, for example, describes how both Daisy and her siblings were all asking questions about why she was taking drugs.

I eventually told her because she asked why she took the drugs alone (Daisy’s Uncle).

This curiosity put caregivers in a difficult position, and they were compelled to first disclose to the HIV-positive young people themselves, then also to the other household members. In their minds, having to tell so many people in quick succession dramatically loosened their control over the information and compounded the risks of unwanted disclosures. This highlights the tension and tussle in giving young people information about their own health, but also the prevailing reticence that imbued caregivers’ attitudes toward disclosing.

However, we found that one caregiver reported telling his child their HIV status directly the first time that she asked him about it. What is unusual about this case is that we were told that Leah had not asked her father any questions about why she was taking treatment until she was 13 years old, which is much later than the rest of the young people and caregivers in our study. By the time she asked, Leah’s father was confident that she understood what HIV was and what it might mean to be HIV positive. Her level of existing knowledge about the condition meant that answering her question directly by disclosing posed fewer risks to him than has been described by many of the other caregivers.

She first asked me what the drugs that she was taking every day were for so I told her that she was born with HIV (Leah’s dad).

### Young People’s Reactions to Disclosure

As we have described when caregivers disclosed this was normally limited to naming the condition and avoiding any further discussion of the implications of their illness. As most caregivers disclosed to facilitate improved adherence, the narrow function of disclosure was why they were taking medications. Discussions, even years later, often did not encompass what impact it may have on young people’s lives and how this could be managed and supported. While disclosure could give hope by emphasizing control through adherence, as shown the inherited nature of the perinatal acquisition of HIV meant that caregivers feared that disclosure could also disrupt and destroy relationships.

Critically, this was not borne out in our data. Over the course of the interviews when we asked the young people about their feelings toward being told they were HIV positive and whether and how this changed over time, they did not describe feeling resentment toward their parents.

Young people did not express anger or blame toward their parents. They had a fair grasp of the unintentional nature of the onward transmission and many understood that the prevention opportunities through treatment had not been available at the time that they had been born.

I didn’t blame them (parents) that much because even when they were alive I never saw them taking any drugs, but if they had been taking them, while we were not I would have blamed them for having kept silent, but they also didn’t know what was going on (Jack, 20 years old).

Most of the young people in the study could describe their “disclosure event,” recounting in considerable detail. They described their reaction as being terrified, worried, confused and intensely emotional. Yet they were not angry at their parents. In the few instances that they were, they described this as being a temporary reaction, which softened or vanished quickly.

When they (counselor) told me (disclosed) I got shocked but I didn’t take it as a very big issue (Amos, 20 years old—was approximately 14 years old when disclosed to).

However, some felt aggrieved by their caregivers’ ambiguity, which met their initial questions and suspicions. They minded that they had been given partial or inaccurate information about their condition and their acquisition of HIV. They expressed frustration that they were not able to have more candid conversations with their caregivers and have their questions answered. It was not the facts that bothered them, but the silence about them. It was this limited access to care and support that had a psychosocial impact. With no further follow-up discussions, their experience felt lonely and isolating.

And from the moment that I heard that (HIV positive status), my heart stopped beating for a moment and I started crying and tears flowed from my eyes without even knowing I was about to cry. I felt death was next, I felt lied to; I felt that I could not have a proper life (Nelly, 18 years old).

## Discussion

Encouraging appropriate and timely disclosure to young people about their own HIV is a central tenet in the care and management of pediatric HIV (13). Disclosure of HIV diagnosis allows young people living with HIV to participate in making decisions they deem appropriate and to be aware of issues regarding their treatment, care, and sexuality. However, as we have illustrated in this article, as a result of the dilemmas that caregivers are faced with, the process was often condensed into a singular event, which took place either at home or at an HIV care facility in the presence of health-care workers. Our findings both echo current research (9) in this field and introduce new reflections from an analysis of caregivers and young people’s accounts about disclosure.

Our inclusion of care dyads and multiple interviews with young people has enabled us to provide valuable insight into the difference between caregivers’ expectations of the relational fallout of disclosure to young people and how it is experienced by young people themselves (34).

Although fear of the relational consequences of disclosing was a significant feature of caregivers’ accounts, no young people described feeling prolonged anger toward their caregivers. Instead, what appeared to negatively impact their relationships from the young people’s perspectives were the caregivers’ silence, refusal to answer questions or obfuscation about the acquisition, nature, and consequences of their condition (39).

Jointly, our study and current literature (33) highlight that the anticipation of negative ramifications of disclosure caused significant anxieties for caregivers, which need further consideration. Disclosure of HIV to children can be a murky process since it is about communicating about a condition that is lifelong, threatening, stigmatized and has no cure. For biological parents, it also involves bringing their own HIV-related life experiences into the picture in a way that may be unusual in their relationship with their children otherwise (40). A blunt and urgent approach to disclosure, for example, as a result of an adherence crisis, often leaves inadequate time for caregivers to feel prepared to deal with imagined or actual consequences (4, 27).

However, secrecy, concealment, and partial truths are part of the fabric of everyday relationships, and HIV disclosure is no exception (41). There is an ambiguity in caregivers’ silence that relates to their desire to protect young people from worry, and to shield them and their household from the possible impact of discrimination. At the same time, caregivers are trying to avoid addressing the question of transmission, and, as other studies have found (32, 33), fear being blamed and losing status in the eyes of their children. It also reflects the difficulty in engaging in conversations, for example, about the future, which have no easy answers (8).

It is certainly important to underscore how awareness and understanding about their HIV diagnosis helps young people develop their own ways to live with the condition, something we have repeatedly found in interviews with young people linked to this study (7, 36, 38) as well as in our other work (3, 8, 42, 43). However, our other studies also point to the problems created by disclosure as a medicalized moment driven by adherence (7, 38) which does not take into consideration the social and the protective functions of silence as well as the tensions inherent to revelations and truths in the family.

Our findings illuminate the perspectives of caregivers as well as those of young people. A closer analysis of our data shows that pressuring caregivers into initiating conversation about HIV when they are not confident or ready may create long-term challenges for them and for young people because disclosure as a “forced” event will be kept to a minimum. While caregivers try to contain the potential damage of imparting information about HIV, this “bare minimum” approach leaves the young person wondering about the rest. Caregivers may be anxious to curtail the extent of disclosure, avoid follow-up questions, and restrict further discussion. The information about HIV may be imparted only as a matter of treatment adherence and onward infection. At the same time, this pivotal moment of partial revelation may signal to young people something about them is wrong, leaving little room for developing a helpful exchange about HIV as they grow up.

Therefore, the caregivers accounts in our study are an important reminder that young people’s “right to know” should not be pitted against what caregivers’ perceive to be their “duty of care,” which includes but also extends beyond “improving adherence”: it is a balancing act between what they see as best for their children (those with and without HIV), themselves and their households. At the same time, caregivers’ desire to minimize harm to their relationship with their children by delaying disclosure might backfire if we take into consideration that young people discuss delays and silences on the matter of their HIV as frustrating and confusing omissions.

Based on our findings from this and other studies with young people, we would like to recommend that disclosure should be a supported process for all involved (6, 9, 44). Caregivers should be encouraged and supported to work toward the point of initiating a planned disclosure process, to avoid disclosure being conducted urgently in response to an adherence crisis. Even if disclosure is reactive, discussions with young people regarding the necessity of medication to sustain health need to be accompanied by a willingness to engage with young people in conversations about resilience and about the kind of present and future life they can envisage and imagine for themselves, to which medication is the means (38).

It is not sufficient to emphasize the necessity of disclosure to caregivers and expect them to be able to act without support. Short, supported disclosure courses should be offered within clinics by counselors to individual or groups of caregivers (45, 46). These should include the following elements. First, caregivers need to be persuaded by the value in the child knowing about their own diagnosis, beyond managing or containing the particular events that they are dealing with at that immediate time. Second, caregivers need to be supported in feeling confident about their own knowledge about HIV, the circumstances of their child’s infection, and the realities of risks and opportunities for prevention of HIV. They also need to feel able to draw on the support of clinic staff in helping them to answer the range of questions that full disclosure may provoke. Third, and this is a point that has to date received relatively little emphasis, caregivers need reassurance that many young people, once they find out their status, do not harbor resentment toward their parents for their own infection. Fourth, caregivers and young people need support in finding strategies to manage potential discrimination. This could be delivered through follow-up meetings for groups of caregivers to offer and benefit from peer support and mentorship.

The relatively small study sample is a limitation for our study. It means that we should be cautious about interpreting gendered patterns in the different approaches of the caregivers. In addition, the data rely on the recall of many of our participants rather than exploring how disclosure was experienced in time. However, this approach provides valuable reflections and insight into the experience of disclosure over time and its effects on the relationships between caregivers and young people.

## Conclusion

Our findings indicate that there is need to actively engage and equip parents and caregivers’ of young people living with HIV with adequate knowledge, information and skills which will prepare them to initiate and facilitate discussions around disclosure and HIV.

## Ethics Statement

This study was carried out in accordance with the recommendations of the Uganda National Council for Science and Technology, National Drug Authority and the Joint Clinical Research Centre Institutional Review Board, with written informed consent from all subjects. In addition to caregivers’ written consent, assent was sought from all participating young people. All subjects gave written informed consent in accordance with the Declaration of Helsinki. All young people were aware of their HIV status before taking part in the study. The protocol was approved by the Uganda National Council for Science and Technology, National Drug Authority, and the Joint Clinical Research Centre Institutional Review Board. The protocol was also approved by the London School of Hygiene and Tropical Medicine. Audio recorded data were transcribed verbatim, anonymized, and translated into English where necessary. To ensure confidentiality of the study participants, only pseudonyms are used in this article.

## Author Contributions

SN conducted fieldwork and data analysis and drafted the article; SP managed fieldwork and collaborated on data analysis and manuscript writing; JS managed the research study and fieldwork and collaborated on data analysis and manuscript writing; SB managed the research study and fieldwork, and collaborated on data analysis and manuscript writing.

## Conflict of Interest Statement

The authors declare that the research was conducted in the absence of any commercial or financial relationships that could be construed as a potential conflict of interest.
